# Exploring the Causality Between Mood Swings and Neurological Diseases: A Mendelian Randomization Study

**DOI:** 10.1002/brb3.71506

**Published:** 2026-05-29

**Authors:** Na Li, Fei He, Shanling Xie, Zheng Wang, Jie Tan, Si Yuan

**Affiliations:** ^1^ South China Research Center for Acupuncture and Moxibustion, Medical College of Acu‐Moxi and Rehabilitation Guangzhou University of Chinese Medicine Guangzhou Guangdong China; ^2^ College of Acupuncture, Massage and Rehabilitation Hunan University of Chinese Medicine Changsha Hunan China

**Keywords:** causal relationship, Mendelian randomization, mood swings, neurological diseases

## Abstract

**Background:**

Associations between genetically predicted mood swings and neurological diseases have been suggested. However, the causal relationships between these factors remain undefined.

**Method:**

We used a Mendelian randomization (MR) study to examine the causal relationship of genetically predicted mood swings with the risk of neurological diseases, including stroke, Alzheimer's disease (AD), multiple sclerosis (MS), epilepsy (EP), and headache. The single‐nucleotide polymorphisms (SNPs) that exhibited genetic associations with mood swings were utilized as instrumental variables (IV) in the study. We performed MR analyses using the inverse variance weighted (IVW) method as the main approach. Sensitivity analyses were further performed using MR‐Egger and MR‐PRESSO to assess the robustness.

**Results:**

The MR analysis revealed significant causal of mood swings on stroke (OR = 1.300, 95% CI = 1.060–1.600; *p* = 0.012), headache (OR = 1.020, 95% CI = 1.000–1.030; *p* = 0.005), excluding AD (*p* = 0.548), EP (*p* = 0.449), MS (*p* = 0.494).

**Conclusion:**

The findings of this research indicate a possible causal link existing between mood swings and stroke and headache. Additional investigative efforts are requisite for elucidating the underlying biological mechanisms that drive these observed associations.

## Graphical abstract

A new exploration to utilize GWAS summary data to evaluate the causal relationship between mood swings and neurological diseases. An affirmative causal link was discerned between fluctuations in mood and the occurrence of stroke as well as headache.

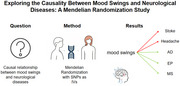

## 1 Introduction

Neurological conditions encompass disorders affecting the central, peripheral, and autonomic nervous systems, leading to impairments in movement, cognitive functions, and pain, all of which can profoundly and adversely affect an individual's overall quality of life (Nong et al. 2023). They include stroke, Alzheimer's disease (AD), epilepsy (EP), Parkinson's (PD), vascular dementia (VD), multiple sclerosis (MS), headaches, and many others. Neurological diseases have emerged as a primary contributor to mortality and disability globally, particularly imposing a significant toll on low‐ and middle‐income nations (Feigin et al. 2020). Neurological diseases pose challenges and potential crises to human health and remain a major threat (GBD 2016 Stroke Collaborators 2019; Zhang et al. 2024). Control of related risk factors is an important means to prevent and treat neurological diseases.

Mood swings refer to sharp changes in the emotional state experienced by an individual over a short period, which usually involves significant fluctuations in the intensity, duration, and nature of the mood, and includes mood states such as irritability, sudden euphoria, anxiety, and depression (Fristad 2022). Although mood swings do not directly equate to mental illness, they are often an important indicator or warning sign of health conditions. In recent years, the intensive examination of the interplay between biological, psychological, and social determinants in disease progression has led to a growing body of evidence emphasizing the pivotal role of mental disorders (MDs) as potential precursors or risk factors for the development of neurological disorders (Nilsson and Kessing 2004; Rezaee et al. 2024). Several studies have conclusively demonstrated that individuals suffering from mental health conditions exhibit a heightened vulnerability to suicide, stroke, and heart attack compared to the broader population (Jackson et al. 2020; Liao et al. 2024).

However, the relationship between the nervous system and mood swings has not yet been studied. The entirety of current evidence stems solely from observational studies, which, while capable of investigating potential disease origins, are inherently prone to confounding variables and the possibility of reversed causality. Uncovering the causal link between mood swings and neurological diseases can offer valuable insights and direction for the prevention strategies and therapeutic approaches aimed at addressing neurological diseases. Therefore, we performed a Two‐Sample MR study to evaluate the potential causal association between mood swings and neurological diseases.

Mendelian randomization (MR) analysis is an epidemiological methodology designed to probe into potential causal relationships between exposures and outcomes. This approach utilizes single nucleotide polymorphisms (SNPs) as surrogate markers or instrumental variables, thereby facilitating the investigation of the exposure of interest. Traditionally, randomized controlled trials hold the position of the gold standard in causal inference. Nonetheless, the execution of these trials is frequently hindered by ethical and legal barriers. MR presents a viable solution to these limitations, offering an alternative approach. The random distribution of genetic variants in MR studies ensures their independence from confounding variables, effectively attenuating the influence of such factors. This is rooted in the fundamental principle that an individual's genotype is not influenced by the presence or absence of a disease (Lawlor et al. 2008). In this investigation, we have compiled genome‐wide association study (GWAS) data to genetically dissect and elucidate the potential causal link between mood fluctuations and five distinct neurological disorders, employing two‐sample MR analyses. Our findings aim to contribute evidence‐based insights for the prevention and management strategies of mood disturbances that may arise in individuals with neurological conditions.

## 2 Materials and Methods

### 2.1 Study Design

**FIGURE 1:**
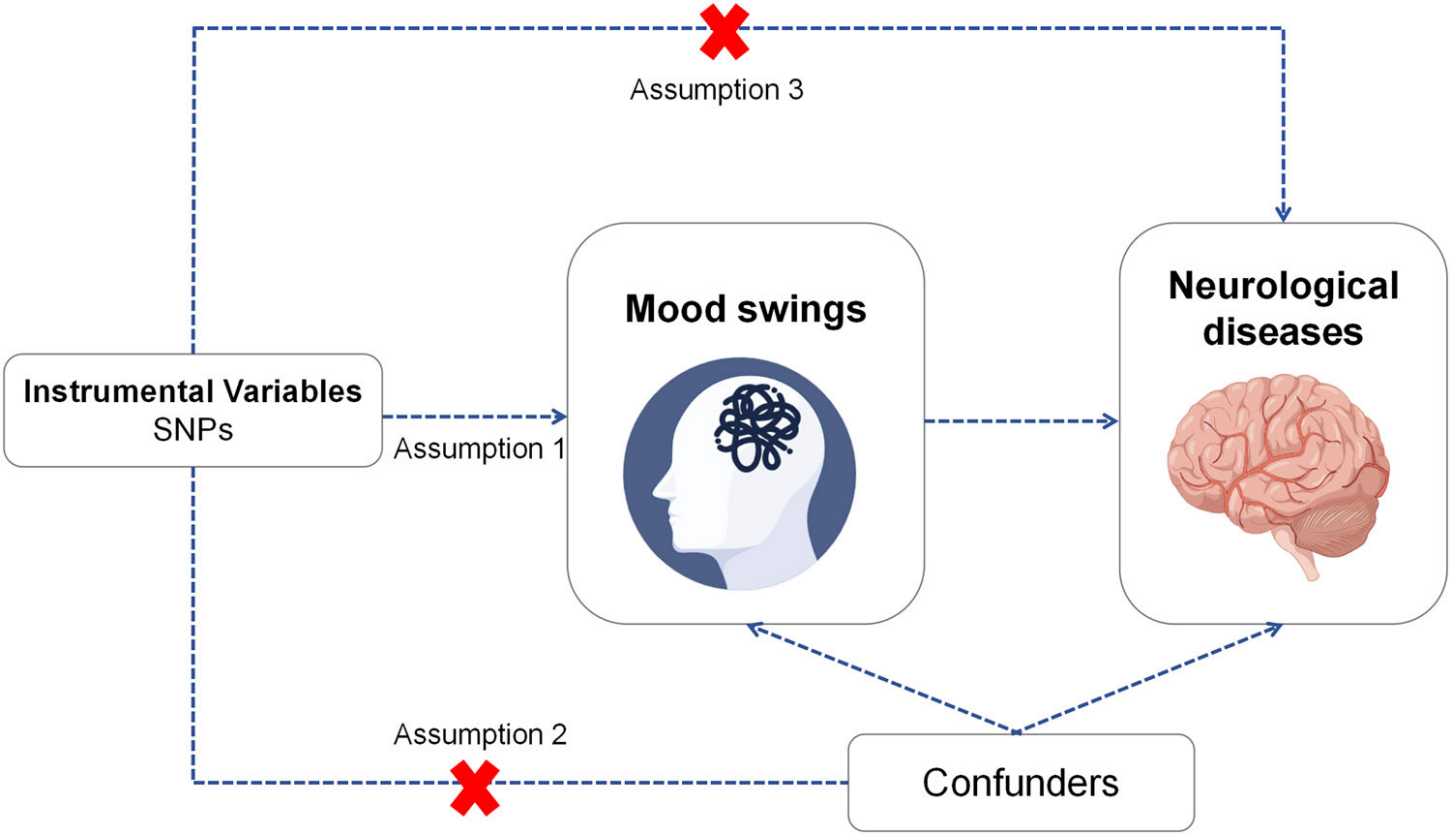
Mendelian randomization study workflow to comprehensively investigate the causal relationship between mood swings and neurological diseases. The design hypotheses are that genetic variants are associated with mood swings, but not with confounders, and the genetic variants are associated with risk of neurological diseases only through mood swings. SNPs, single nucleotide polymorphisms.

We conducted a two‐sample MR analysis to assess the potential causal relationship between mood swings and neurological diseases. The MR analysis integrated the cumulative effects of a series of SNPs, which are strongly associated with exposure variables, on the incidence of outcome events. This comprehensive evaluation was undertaken to clarify the causal relationship between the exposure factors and the outcomes. In this approach, mood swings are the exposure variables, and SNPs strongly related to mood swings were utilized as IVs. The outcomes included five different neurological diseases: stroke, AD, EP, MS, and headaches. To reliably establish the causal relationship between exposure and outcome in MR analyses, three fundamental assumptions must be met: First, the genetic variation must exhibit a robust association with the exposure of interest. Second, this genetic variation should remain uncorrelated with any potential confounding variables. Lastly, the genetic variation should solely influence the outcome through its relationship with the exposure, excluding any direct or indirect effects independent of the exposure (Emdin et al. 2017). The analytical procedure is depicted in Figure 1.

### 2.2 Data Sources

GWAS summary data were used for MR analysis in our study. All data were obtained from the original study of the IEU OpenGWAS project (https://gwas.mrcieu.ac.uk). The mood swings data used in this paper originates from an original study designed as a genome‐wide association study (GWAS). Throughout this manuscript, the term “mood swings” refers specifically to this genetically proxied trait of frequent mood fluctuations. It does not represent clinically diagnosed mood swings. This study employed a questionnaire instrument to measure the psychological trait of neuroticism, with emotional volatility—as a facet of neuroticism—also being indirectly assessed through this questionnaire. Mood swings data encompassed a collection of 10,824,841 individuals of European descent (373,733 cases and 10,451,108 controls). To counteract potential biases stemming from population stratification, the GWAS dataset employed in our study was confined exclusively to samples from European populations. Given that this research leveraged data sourced from publicly accessible databases and previously published studies, it was deemed exempt from the requirement for additional ethical approval or informed consent. This adherence aligns with the ethical principles and guidelines that governed the original studies where these data were originally collected and analyzed. Table 1 presents details about the datasets.

**TABLE 1:** Source of the GWAS data.

Traits	Year	Race	Sample size (cases/controls)	Datasets in the GWAS
Mood swings	2018	European	373,733/10,451108	ebi‐a‐GCST006944
IS	2018	European	34,217/406,111	ebi‐a‐GCST005843
AD	2022	European	39,106/46,828	ebi‐a‐GCST90027158
EP	2021	European	1781/212,532	finn‐b‐GE
MS	2019	European	47,429/68,374	ieu‐b‐18
Headache	2021	European	4,122/480,476	ebi‐a‐GCST90038675

IS, ischemic stroke; AD, Alzheimer's disease; EP, generalized epilepsy; MS, multiple sclerosis.

### 2.3 Instrument Selection

We searched the IEU OpenGWAS project for summary data on exposure‐related GWAS SNPs. To minimize potential bias from linkage disequilibrium among SNPs, stringent screening criteria were used (Thomas and Conti 2004): (1) For data used as exposures, SNPs with a significance level of *p* <5×10^−8^ were used. (2) SNPs with linkage disequilibrium between selected IVs were excluded, with the threshold set at *r* ^2^ < 0.001 and kb = 10,000. (3) From the resulting GWAS data to retrieve relevant information about the selected IVs, conduct allele alignment to ensure consistency, and eliminate palindromic sequences that exhibit intermediate allele frequencies. (4) We calculate the *F*‐value using the following formula: *F* = β^2^/SE^2^. Ensure all *F*‐values exceed 10, indicating that the instrumental variables are unaffected by weak instrumental variable bias.

### 2.4 MR Analysis

The entirety of the statistical analyses conducted in this study was executed utilizing the “Two Sample MR” packages within the RStudio environment. This study included five different techniques: inverse variance weighted (IVW), MR‐Egger regression, weighted median, weighted mode, and simple mode. The robustness of the results is tested through these five methods (Shu et al. 2022). The IVW methodology computes the averaged, weighted influence of the effect sizes across the entire spectrum of IVs throughout the analysis, resulting in comparatively reliable outcomes (Burgess et al. 2013). Based on the effect estimate of each instrumental variable and its variance, effect estimates are assigned weights so that instrumental variables with lower variance have greater weights. Therefore, IVW results were chosen as the main indicator for assessing causal effects, while other methods were used for complementary assessments (Zhou et al. 2024). Results are provided as 95% confidence intervals (CIs) and ratio of ratios (ORs), with *p* < 0.05 indicating statistical significance.

To examine the direction of causality, we performed bidirectional MR analyses by swapping the exposure and outcome. Specifically, we used each neurological disease (stroke, Alzheimer's disease, multiple sclerosis, epilepsy, and headache) as the exposure and mood swings as the outcome. The same instrumental variable selection criteria and analytical methods (IVW as the primary method) were applied.

### 2.5 Sensitivity Analysis

A series of rigorous sensitivity analyses were conducted to assess the stability and reliability of the results obtained. In this study, potential heterogeneity between instrumental variables at each analysis was assessed using Cochran's *Q* test (Bowden et al. 2015), with a *p* > 0.05 representing no significant heterogeneity problem. Also, to validate the dependability of the findings, the MR‐Egger intercept test was used for directional bias due to the polyvalence of genetic variants, and a directionality of *p* > 0.05 would indicate that there is no horizontal polyvalence problem. Additional leave‐one‐out analyses were conducted for a visual evaluation of the impact individual SNPs have on causal robustness (Ruan and Zhao 2024).

## 3 Results

### 3.1 Causal Effect of Mood Swings on Stroke, AD, EP, MS, and Headache

**FIGURE 2:**
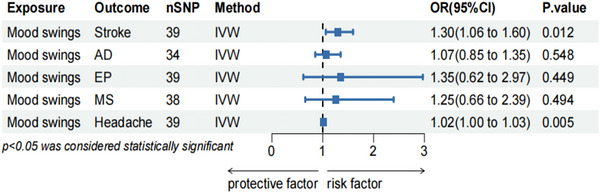
Using inverse variance weighted method (IVW) to estimate the association between mood swings and neurological diseases. nSNP, number of single nucleotide polymorphisms; OR, odds ratio; CI, confidence interval. AD, Alzheimer's disease; EP, generalized epilepsy; MS, multiple sclerosis.

**FIGURE 3:**
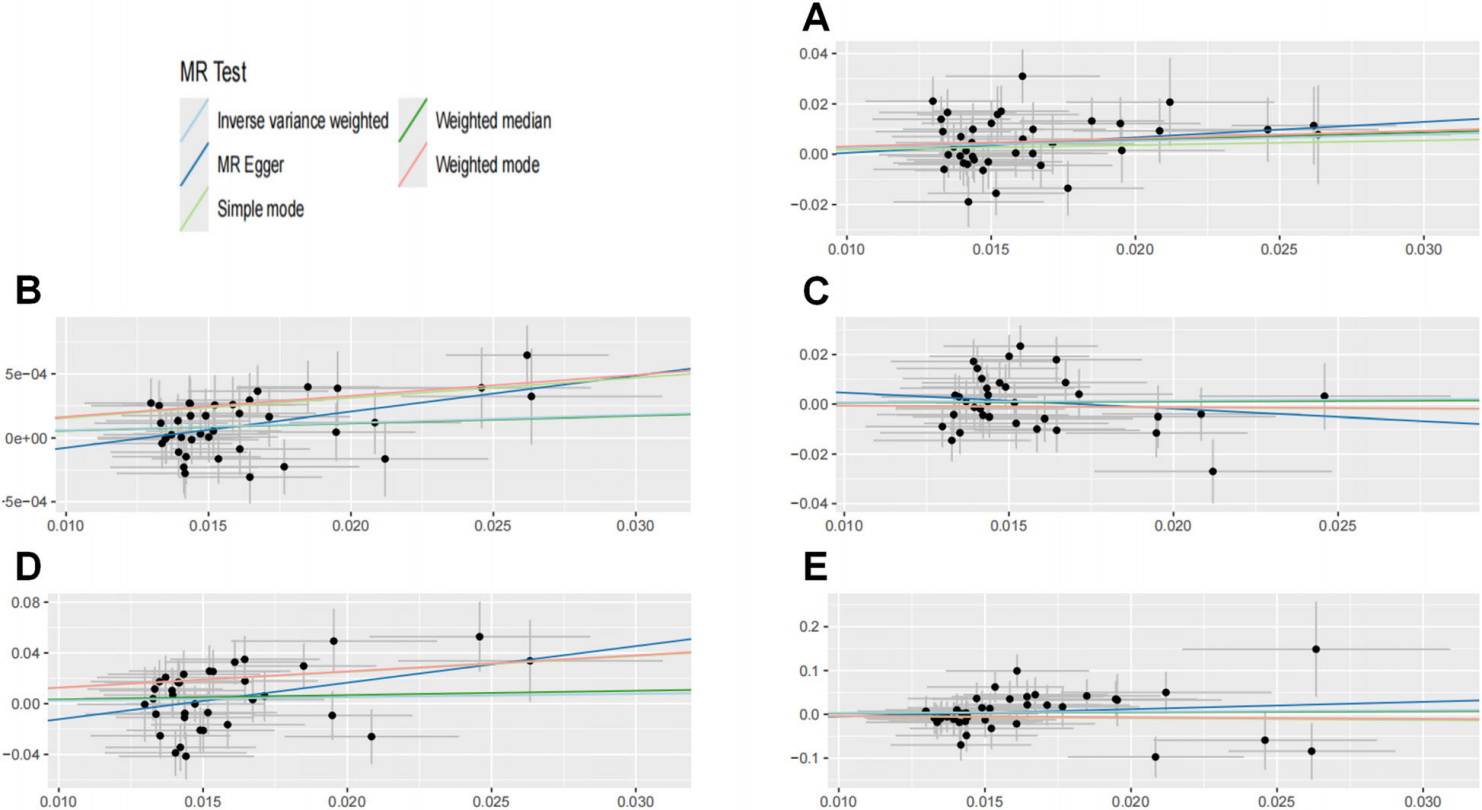
Scatterplot of single nucleotide polymorphisms (SNPs) associated with mood swings and neurological disorders. The slope of each line represents the causal relationship for each method. A: stroke; B: headache; C: AD; D: MS; E: EP.

First, we embarked on an investigation to examine the causal relationship between mood swings and the incidence of stroke, AD, EP, MS, and headache. The IVW analysis results showed an association between mood swings and stroke (OR = 1.300; 95% CI: 1.060–1.600, *p* = 0.012) and headache (OR = 1.020; 95% CI: 1.000–1.030, *p* = 0.005). No causal relationship was identified between mood swings and AD, EP, and MS (Figures 2 and 3). We also attempted to repeat the MR analysis using other databases. Similar results were observed between mood swings and five cardiovascular diseases.

To test the possibility of reverse causation, we conducted bidirectional MR analyses using each neurological disease as the exposure and mood swings as the outcome (Table 2), the reverse MR analyses revealed no significant causal effect of any neurological disease on mood swings(*p* >0.05)

**TABLE 2:** Bidirectional MR results (IVW method).

Exposure	Outcome	*p*‐value
**Stoke**	Mood swings	0.9741680
**AD**	Mood swings	0.7349758
**EP**	Mood swings	0.948174
**MS**	Mood swings	0.2341204
**Headache**	Mood swings	0.5194447

AD, Alzheimer's disease; EP, generalized epilepsy; MS, multiple sclerosis.

### 3.2 Sensitivity Analysis

**FIGURE 4:**
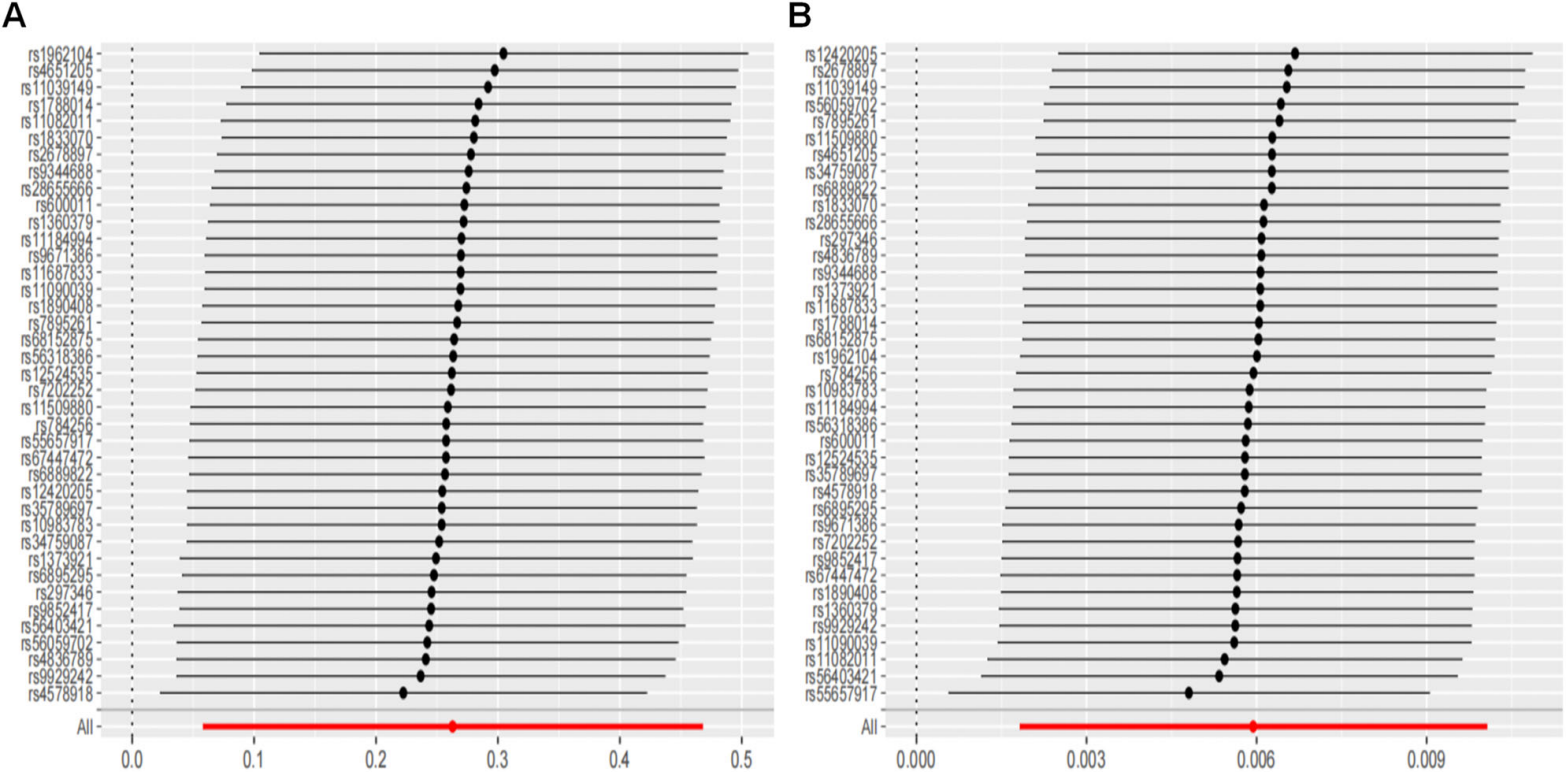
Leave‐one‐out sensitivity analysis between mood swings and neurological diseases, the leave‐one‐out graphs indicated that all lines were located to the right of the “0 line,” indicating the removal of any SNPs had no fundamental effect on the results, suggesting that the MR results were reliable. A: stroke; B: headache.

Furthermore, to supplement our examination of the causal linkage between mood fluctuations and the aforementioned five conditions (stroke, headache, AD, EP, and MS), we performed rigorous sensitivity analyses utilizing three distinct tests. The pleiotropy examination revealed that all the analyses failed to indicate the existence of pleiotropic effects (Table 3). The leave‐one‐out analysis revealed that the observed causal effect was not attributable to any single instrumental variable, suggesting robustness and independence from any singular influencing factor (Figure 4). Figures S1 and S2 display the funnel plot and leave‐one‐out plot of AD, EP, and MS. Figure S3 displays the funnel plot of stroke and headache.

**TABLE 3:** Heterogeneity and pleiotropy test for the results of MR analysis of causal relationships between mood swings and neurological disorders.

			Pleiotropy test		Cochran's *Q* test		
			MR‐Egger		MR‐Egger		
Exposure	Outcomes	Intercept	SE	*p*‐value	*Q*	*Q*‐*p*val	MR‐PRESSO
Mood swings	Stroke	−0.0056	0.0100	0.5793	40.6674	0.3121	0.3220
Mood swings	AD	0.0111	0.0129	0.3972	44.6009	0.0685	0.0700
Mood swings	EP	−0.0216	0.0398	0.5912	35.4795	0.5404	0.5650
Mood swings	MS	0.0039	0.0307	0.8993	47.6717	0.0473	0.8990
Mood swings	Headache	−0.0004	0.0002	0.0662	32.3603	0.6863	0.5610

AD, Alzheimer's disease; EP, generalized epilepsy; MS, multiple sclerosis.

## 4 Discussion

This study is a new exploration to utilize GWAS summary data to evaluate the causal relationship between mood swings and neurological diseases. This analysis yielded two crucial insights. First, an affirmative causal link was discerned between fluctuations in mood and the occurrence of stroke as well as headache, implying that such mood variations could potentially elevate the likelihood of developing both aforementioned medical conditions. Second, the analysis failed to uncover any statistically significant causal association between mood swings and AD, EP, and MS.

Research has demonstrated that fostering positive mental health can contribute to a decrease in the occurrence of strokes (Lambiase et al. 2015). A comprehensive multi‐racial GWAS will enhance the productivity of future MR studies to clarify the intricate relationship between mood swings and neurological diseases (Shen et al. 2024). A recent MR study has provided evidence suggesting that mood swings may elevate the likelihood of developing cardiovascular diseases (Liu et al. 2024). Another MR analysis suggested that psychiatric traits such as insomnia and emotional instability have a risk effect on intracranial aneurysms (IA) (Peng et al. 2023). However, a dearth of systematic investigations exists in elucidating the potential causal linkage between mood swings and a range of neurological disorders. There are many known risk factors for neurological disorders, and identifying new, nontraditional risk factors or preventable triggers is becoming increasingly critical to developing more targeted and detailed prevention strategies.

Our discoveries have enriched this domain by establishing a definitive causal link between mood fluctuations and neurological disorders, offering fresh perspectives on their relationship. Given the multitude of identified influences and risk factors associated with neurological disorders, our study delves into novel factors that are pivotal in the onset and progression of these conditions. Nevertheless, the precise pathophysiological pathways through which mood fluctuations exert an impact on the advancement of specific neurological conditions remain elusive. Future research endeavors will concentrate on elucidating the precise mechanism that underlies the observed causal relationship and exploring treatment aimed at alleviating mood fluctuations.

We postulate several plausible hypotheses to illuminate the mechanisms underlying the contribution of mood swings to the initiation and advancement of neurological diseases. First, when emotions are chronically dysfunctional, the brain releases different neurotransmitters such as dopamine (Delva and Stanwood 2021) and 5‐hydroxytryptophan (Tejeda‐Martínez et al. 2024), and changes in the levels of these neurotransmitters can affect the stability and function of the nervous system. Second, emotions such as anxiety and depression cause the sympathetic nervous system to become over‐activated, releasing large amounts of stress hormones. Research has indicated a correlation between variations in cortisol levels and a multitude of psychiatric conditions (Patel et al. 2024), including anxiety and depression (Druzhkova et al. 2022). Elevated levels of glucocorticoids may potentially lead to ischemic neuronal injury and augment the likelihood of developing neurological disorders (Gold et al. 2005). Dysregulation of the hypothalamic‐pituitary‐adrenal (HPA) axis can elicit disorders of the autonomic nervous system's sympathetic branch, structural modifications in brain architecture, and cognitive deficiencies (Vyas et al. 2023). Third, the inflammatory response constitutes a crucial aspect of the body's defensive mechanism against diverse stressors (Liu et al. 2024), encompassing both psychological stress and specific physical stimuli. An excessive inflammatory response may cause damage to nerve tissue, which can trigger or exacerbate neurological disorders (Libby et al. 2009). In addition, chronic emotional instability may contribute to behavioral patterns such as lack of regularity (Lian et al. 2024), poor diet (Rabat et al. 2021), excessive dependence on stimulants (Mozzini et al. 2016), and reduced physical activity (Andrade 2024), all of which have been empirically established to correlate with an elevated likelihood of developing neurological disorders.

Undoubtedly, there are inherent constraints in this study. First, despite MR offering a more robust level of evidence than conventional epidemiological approaches, it falls short of being a substitute for randomized controlled trials, which remain the pinnacle of research standards. In MR studies, effectively eliminating the influences stemming from potential pleiotropic effects poses a formidable challenge. Consequently, our deductions must be interpreted within the broader framework established by the findings of rigorously conducted randomized controlled trials, ensuring a nuanced and comprehensive assessment of our conclusions. Second, the exposure factor is a genetic prediction of emotional fluctuations and has not been clinically validated. Therefore, our research results reflect the trait‐level tendencies of emotional fluctuations rather than the clinical‐diagnosed episodes of emotional fluctuations (such as the episodes in bipolar disorder). However, this operational definition has been used in several published Mendelian randomization studies; the magnitude and prevalence of mood fluctuations require additional validation; and our research was inherently limited by the availability of relevant mood swing data within the genetic databases, thereby restricting our ability to conduct a more targeted and comprehensive exploration. Third, the GWAS study was conducted mainly in European populations, so generalizability is an issue, and validation in ethnically diverse populations is a critical step that needs to be implemented in subsequent studies. Finally, a potential limitation of the current study lies in the lack of multiple data validations, as our conclusions are primarily based on Mendelian randomization analysis. Future research could incorporate biological evidence, such as findings from relevant animal models and clinical data, to cross‐validate existing results and enhance their reliability.

In summary, the implications of our discoveries are manifold, offering valuable insights to both public health and education domains by emphasizing the pivotal role of individual emotional well‐being in mitigating the risk of certain neurological disorders, thereby informing strategic interventions and policies. By elucidating the intricate connection between mental health and neurological disorders, we can foster enhanced awareness and facilitate earlier interventions, ultimately contributing to a decrease in both the prevalence and severity of these conditions.

## 5 Conclusion

In this study, we investigated the causal relationship between mood swings and neurological diseases based on a European population using a two‐sample MR analysis. In conclusion, our two‐sample MR analysis has uncovered a causative association between mood swings and both stroke and headache. However, further investigations are warranted to decipher the intricate mechanisms that underlie the diverse causal linkages observed between mood swings and distinct subtypes of these neurological conditions.

## Author Contributions

**Si Yuan**: writing – review and editing. **Shanling Xie**: visualization. **Fei He**: investigation. **Jie Tan**: writing – review and editing. **Na Li**: writing – original draft, visualization. **Zheng Wang**: data curation.

## Funding

This study was supported by the Young Scientists Fund of the National Natural Science Foundation of China (No. 82405591), Key project of Hunan University of Chinese Medicine (No. Z2023XJZD08), General Project of Hunan Traditional Chinese Medicine Research Project (No. B2024011), Hunan University of Chinese Medicine Undergraduate Research Innovation Fund Project (No. S202410541039).

## Conflicts of Interest

All authors report no conflicts of interest in this work.

## Competing Interests

The authors declare no competing interests.

## Supporting information




**Supplementary Information**: brb371506‐sup‐0001‐figureS1.png

**Supplementary Information**: brb371506‐sup‐0002‐figureS2.png

**Supplementary Information**: brb371506‐sup‐0003‐figureS3.png

## Data Availability

The datasets can be accessed by the interested parties upon submitting a reasonable request to the corresponding author.
