# Supplementary figures and images for: Exploring the Causality Between Mood Swings and Neurological Diseases: A Mendelian Randomization Study
Source: Brain Behav. 2026 May 29;16(6):e71506. doi: 10.1002/brb3.71506 (PMC13239466; doi:10.1002/brb3.71506)

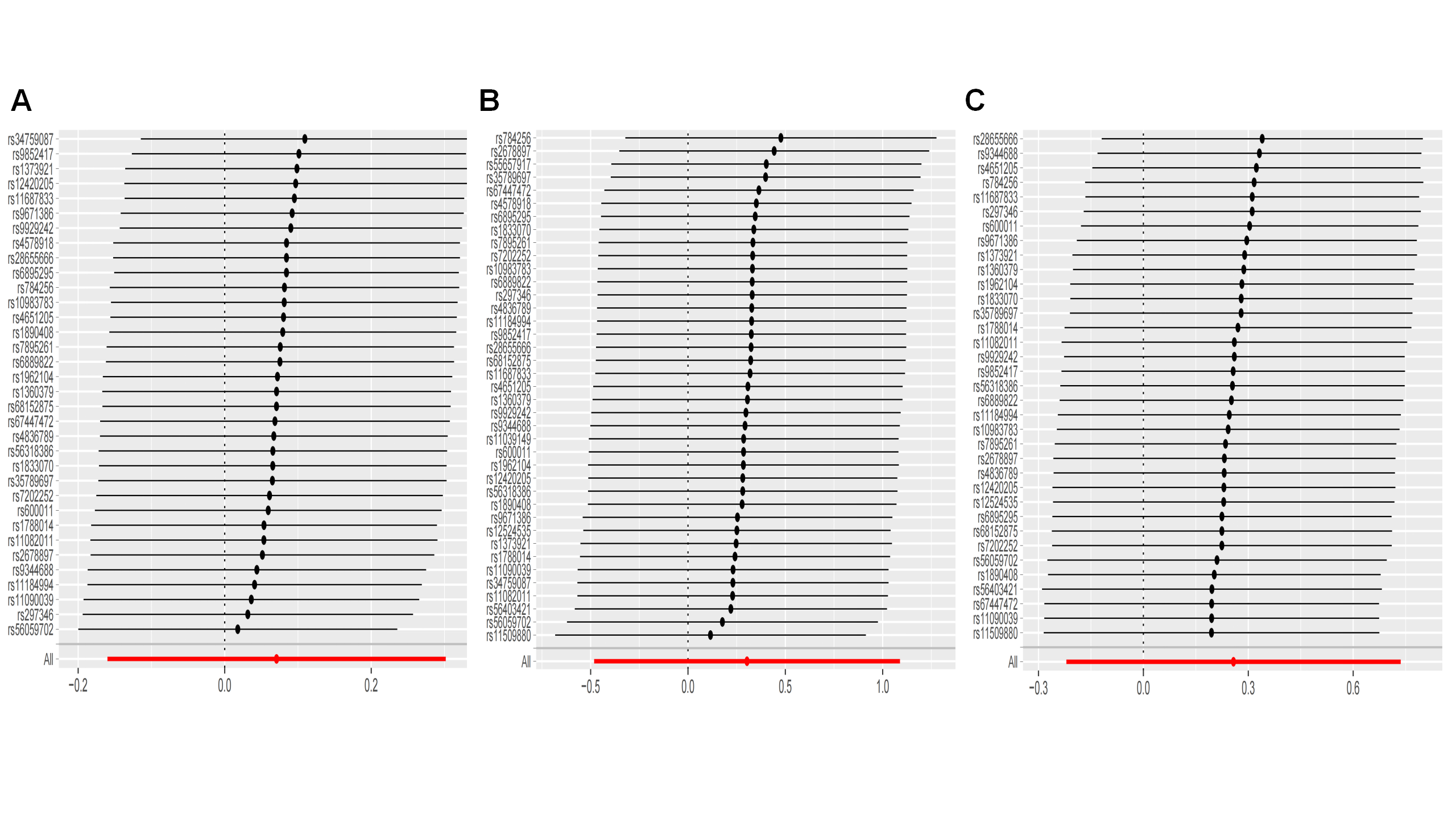

Supplement: Supplementary file 1 — Supplementary Information: brb371506‐sup‐0001‐figureS1.png [file BRB3-16-e71506-s001.png]

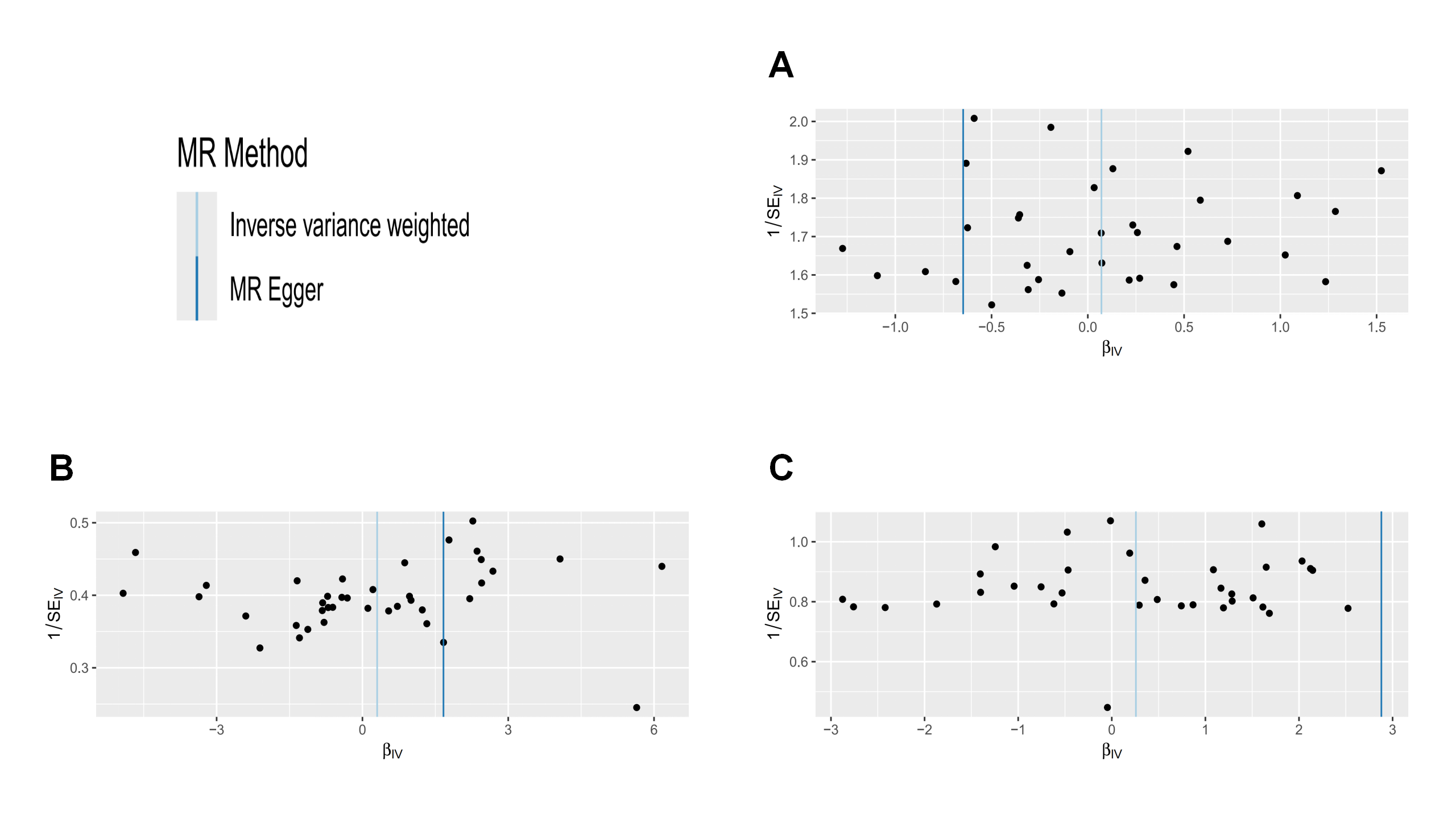

Supplement: Supplementary file 2 — Supplementary Information: brb371506‐sup‐0002‐figureS2.png [file BRB3-16-e71506-s002.png]

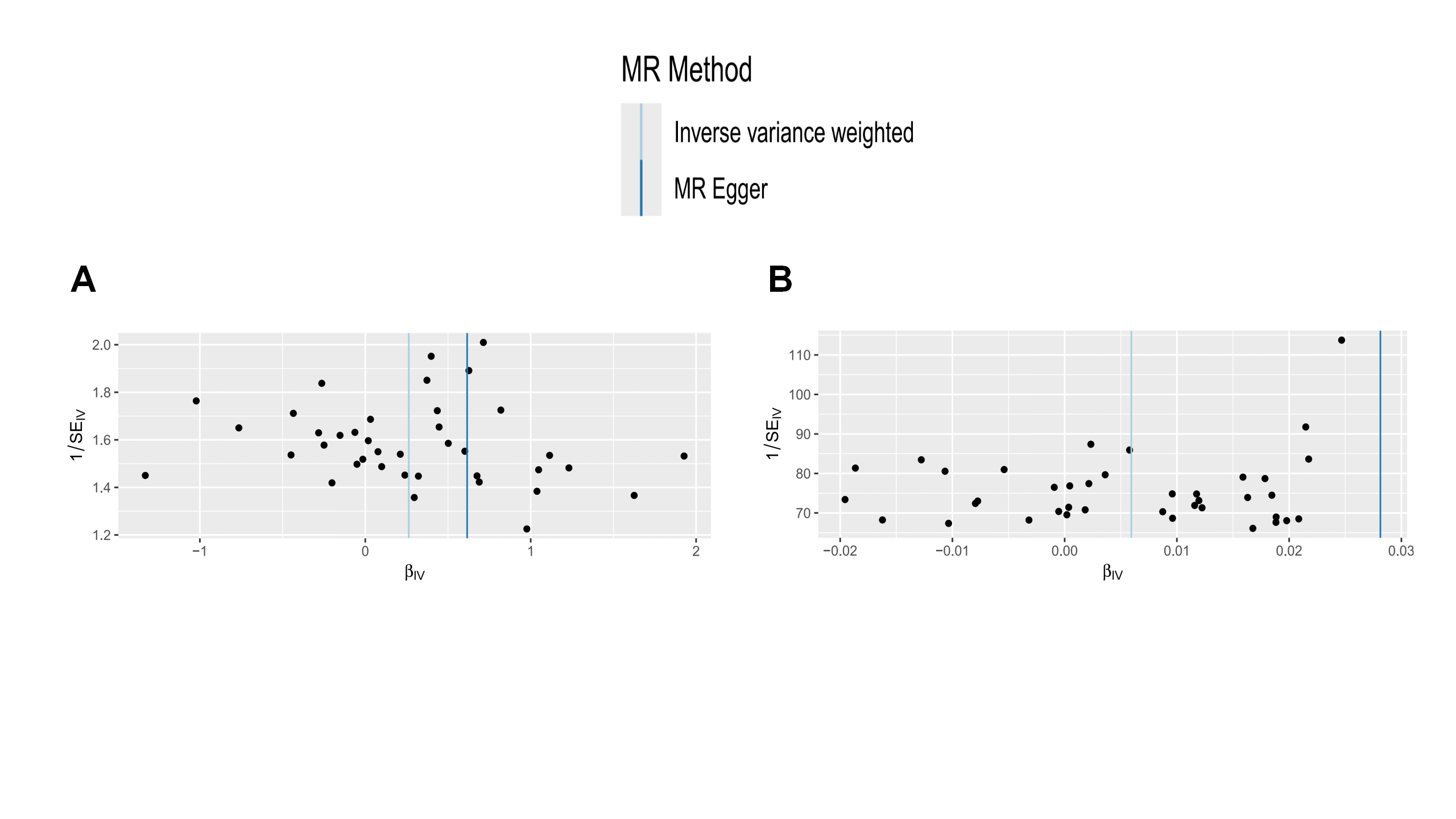

Supplement: Supplementary file 3 — Supplementary Information: brb371506‐sup‐0003‐figureS3.png [file BRB3-16-e71506-s003.png]
